# Considering Mycological Rarities

**DOI:** 10.3201/eid2709.AC2709

**Published:** 2021-09

**Authors:** Byron Breedlove

**Affiliations:** Centers for Disease Control and Prevention, Atlanta, Georgia, USA

**Keywords:** art science connection, emerging infectious diseases, art and medicine, about the cover, considering mycological rarities, Mattia di Nanni di Stefano, Scipio Africanus, mycology, microbes, fungi, fungal infections, Candida auris

**Figure:**
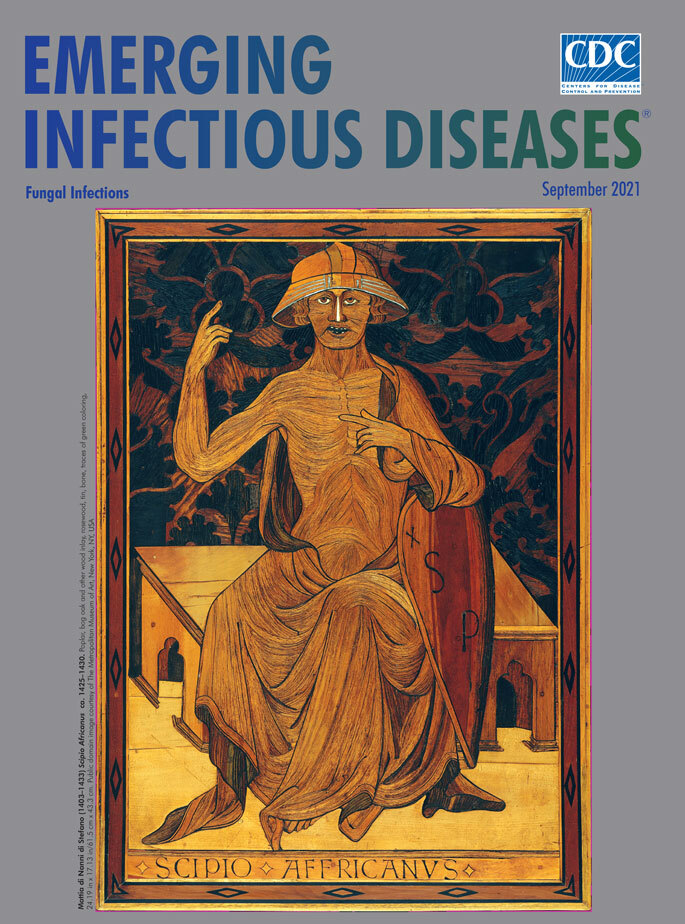
**Mattia di Nanni di Stefano (1403–1433), Scipio Africanus (ca. 1425–1430).** Poplar, bog oak and other wood inlay, rosewood, tin, bone, traces of green coloring. 24.19 in x 17.13 in/61.5 cm x 43.3 cm. Public domain image courtesy of The Metropolitan Museum of Art, New York, NY, USA.

Neither plant nor animal, fungal organisms―including lichen, mildew, mushrooms, molds, rusts, smuts, and yeasts―are found in nearly every possible terrestrial habitat, even aboard the International Space Station. There are millions of species of fungi, and according to the Centers for Disease Control and Prevention, a few hundred fungal species cause illness in people, ranging from allergies and asthma, to skin rashes and infections, to deadly infections of the bloodstream or lungs. 

In a 2013 EID article, Mary Brandt and Benjamin Park note the growing number of human infections from traditional and new fungal agents. Factors driving this emergence, they explain, include medical treatments that make immunocompromised patients more susceptible. They also state that “Risk factors such as changes in land use, seasonal migration, international travel, extreme weather, and natural disasters, and the use of azole antifungal agents in large-scale agriculture are believed to underlie many of the increases in community-acquired fungal infections.” 

The recent emergence of *Candida auris* infections, for instance, underscores those concerns on a broad scale because *C. auris* is often multidrug-resistant, difficult to identify, and causes outbreaks in healthcare settings. A recent study from Finland that reported life-threatening fungal bloodstream infections associated with consuming probiotic supplements that contain *Saccharomyces boulardii* reveals a route of infection that may represent another mycological issue. 

Fungi also have beneficial medicinal and culinary attributes. They were used in traditional medicine long before Alexander Fleming identified and extracted the therapeutic ingredient penicillin from *Penicillium* in 1928. They have subsequently been used to develop antibiotics, fungicides, anticancer drugs, and cholesterol inhibitors. Mushrooms and truffles are highly desirable foods; yeast is essential for baking, brewing, and fermenting; and molds flavor and color cheeses. 

Another attribute of fungi, spalted wood―that is, wood colonized and stained by certain species of fungi―was a prized commodity among European artisans who practiced the form of wood inlaying called intarsia. Spalted wood may be naturally created or stained by an artist; colors may be green, red, yellow, brown, or black. Writer David Elkind explains that green wood discolored by the green elf cup fungus *Chlorociboria aeruginascens* “happened to fill a lucrative niche in a burgeoning luxury trade, and that made it, for a time at least, as precious as some rare metals.” 

Intarsia, described as painting with wood to create mosaics as opposed to painting directly onto wood, is thought to have originated before the seventh century ce. Its zenith was in Italy during the Renaissance (c. 1400−1600). The Tuscan city of Siena, Italy, known for producing many accomplished painters, was home to several *intarsiatori*, including Domenico di Niccolo and his apprentice Mattia di Nanni. *Intarsiatori* inlayed varied shapes, sizes, and species of wood―each with distinct patterns and tones―to fashion decorative items, panels, and elaborate pieces of furniture.

Featured on this month’s cover is a wooden panel depicting Roman general Scipio Africanus, crafted by Mattia. According to the Metropolitan Museum of Art, this panel came from what must have been a quite large intarsia bench created for the council chamber of the Palazzo Pubblico in Siena and placed under Simone Martini's fresco the *Maestà*, a 7.62 m × 9.98 m painting that fills the north wall of the chamber. The bench comprised several panels depicting figures from Roman Republican history considered to be “models of civic virtue, such as the illustrious general Publius Cornelius Scipio Africanus.” Scipio is remembered for the strategic and diplomatic skills that enabled him to defeat Hannibal in the Battle of Zama and end the Second Punic War in 202 bce. 

Mattia portrays Scipio gesturing with his hands―perhaps making the point that a leader must follow his head and his heart―and fixing an unyielding gaze on the viewer. The whorls and details in the interlocked wood pieces show muscles, eyes, hair, a draped tunic. A rich, patterned background adds contrast and texture. Noted woodworker Silas Kopf writes that Mattia’s skills surpassed those of Domenico, from whom he had learned “how to create a strong graphic presentation through contrast, developing the craft further by laminating small pieces of wood into larger shapes.” 

*Intarsiatori* mapped out patterns and colors on paper and then created a matrix or framework to be filled in with different types, shapes, and sizes of wood. Their toolbox included saws, planes, chisels, clamps, knives, pigments, and varnishes. Intarsia projects required large amounts of different colored and textured types of wood, including oak, cypress, walnut, fruitwoods, boxwood, and spindle-wood. The artist would attach sections and pieces of wood called *tesserae* to the frame, following the paper template, incorporating larger pieces, and filling in with smaller ones to add details and depth. Mattia was among those who used additional materials: his *Scipio Africanus* features teeth made from bone and a helmet inlaid with metal strips.

Art historian Antoine Wilmering notes that Mattia meticulously tapered the ends of the *tesserae*, “enabling precise and smooth interweaving of the different, naturally coloured woods. This technique allowed Mattia to create images with carefully modelled details, and some of the inlaid slivers are as fine as a painter's brush.” The greenish tints in this panel may be slivers of naturally spalted wood, likely *griinfaule* or “green oak.” As intarsia expanded across Europe, such wood became highly prized. Elkind notes that green wood discolored by the green elf cup fungus *C. aeruginascens* was “a mycological rarity.”

The craft of intarsia continued to evolve, but spalted wood fell into disfavor once inorganic dyes and stains were readily available. Interest in incorporating spalted wood into intarsia was rekindled in the 1950s, and Professor Sara C. Robinson oversees a laboratory at Oregon State University focused on finding new uses for spalted wood not limited to the creative arts. A recent article by Hyde et. al. in the journal *Fungal Diversity* takes a broader view and examines 50 ways to exploit fungi as an untapped resource, including applications as antibacterials, antimycotics, fungicides, and biofilm inhibitors. Ubiquitous and unique, fungi have a fascinating array of yet unexplored uses.
